# Efficacy of [^68^Ga]Ga-FAPI-PET as a non-invasive evaluation method of liver fibrosis

**DOI:** 10.1007/s12149-025-02027-6

**Published:** 2025-03-06

**Authors:** Yuriko Mori, Katharina Tamburini, Emil Novruzov, Dominik Schmitt, Eleni Mavriopoulou, Sven H. Loosen, Christoph Roderburg, Tadashi Watabe, Clemens Kratochwil, Manuel Röhrich, Abass Alavi, Uwe Haberkorn, Frederik L. Giesel

**Affiliations:** 1 Department of Nuclear Medicine, Medical Faculty and University Hospital Duesseldorf, Heinrich-Heine-University Duesseldorf, Moorenstrasse 5, 40225 Duesseldorf, Germany; 2 Department of Nuclear Medicine, Heidelberg University Hospital, INF 400, 69120 Heidelberg, Germany; 3 Department of Gastroenterology, Hepatology and Infectious Diseases, Medical Faculty and University Hospital Duesseldorf, Heinrich-Heine-University Duesseldorf, Moorenstrasse 5, 40225 Duesseldorf, Germany; 4 Department of Nuclear Medicine, Mainz University Hospital, Langenbeckstraße 1, 55131 Mainz, Germany; 5 Department of Radiology, Hospital of University of Pennsylvania, Philadelphia, PA USA; 6 Institute for Radiation Sciences, Osaka University, 2-2 Yamadaoka, Suita, Osaka 565-0871 Japan

**Keywords:** Fibroblast activation protein, FAPI, PET, Liver, Fibrosis

## Abstract

**Introduction:**

Liver fibrosis is a chronic fibrosing hepatic disorder following recurrent injury, characterized by the excessive accumulation of extracellular matrix. Early detection has a great clinical impact because 80–90% of hepatocellular carcinomas are known to develop in fibrotic or cirrhotic (end-stage fibrotic) livers. PET imaging with FAP ligands exhibited highly promising results in recent years to visualize fibrosis in various organs due to the crucial role of activated fibroblasts in fibrosing processes. However, still little is known about the efficacy of FAP imaging in liver fibrosis. Thus, we sought to investigate the potential of FAPI-PET in a cohort of oncological and non-oncological patients.

**Methods:**

199 patients who underwent FAPI-PET/CT at the University Hospital of Heidelberg between July 2017 and July 2020 were retrospectively analyzed. The tracer uptake of the liver was analyzed and correlated with radiological and clinical parameters.

**Results:**

We observed a weak but significant negative correlation between the hepatic FAPI uptake and CT density (*r* = − 0.273, *P* < 0.001***). A positive correlation was observed between hepatic FAPI uptake and the aspartate aminotransferase (AST)-to-platelet ratio index (APRI) (*r* = 0.183, *P* = 0.009**), an established surrogate for liver fibrosis. The liver SUV (standardized uptake value) mean and SUVmax of FAPI showed significant differences between groups of patients with low (< 0.5), middle (0.5–1.0) and higher (> 1.0) levels of APRI (both *P* < 0.001***).

**Conclusion:**

These preliminary observational results suggest that FAPI-PET may be a viable non-invasive method to asses liver fibrosis.

**Supplementary Information:**

The online version contains supplementary material available at 10.1007/s12149-025-02027-6.

## Introduction

Liver fibrosis is a regenerative tissue process following liver injury and is characterized by an increased extracellular matrix (ECM) deposition [1]. Chronic liver injury such as alcoholic liver disease, non-alcoholic fatty liver disease (NAFLD) or viral hepatitis (chronic hepatitis B or C), can lead to recurrent scarring and subsequent progressive fibrogenic processes, resulting in an abnormal proliferative tissue response [1]. The end-stage disease is known as cirrhosis, which, together with other chronic liver diseases, is the 14th leading cause of death worldwide [2, 3]. Early detection of liver fibrosis and/or cirrhosis has a significant clinical impact, as 80–90% of hepatocellular carcinomas (HCCs) are known to develop in fibrotic or cirrhotic livers [4].

At the cellular level, hepatic stellate cells (HSCs), a vitamin A-storing cell located in the perisinusoidal space (space of Disse), play a predominant role in liver fibrogenesis [5]. Quiescent HSCs can be activated after injury and differentiate into myofibroblasts, which then migrate to the repair site and begin to proliferate, facilitating further fibrogenic processes through active secretion of growth factors and cytokines [6]. Activated HSCs can be distinguished from the inactive quiescent phenotype by the expression of surface markers such as alpha-smooth muscle actin (α-SMA) and fibroblast activation protein (FAP) [7, 8].

The recent introduction of FAP ligands as fibroblast-targeting agents [9–11] offers a potentially effective method of non-invasively detecting fibrosis in various organs at an early stage of disease [12, 13]. Several studies have demonstrated the efficacy of FAP ligands in fibrotic organ processes, such as lung [14] or kidney [15], suggesting the promising potential of FAP imaging to detect the clinical course of fibrosis. The evaluation of liver fibrosis with FAP in a large cohort of patients has not yet been performed. Therefore, we aimed to provide the first preliminary evaluation of hepatic FAP expression in a cohort of oncological and non-oncological patients.

## Material and methods

### Patient cohort

The cohort consists of 199 patients, who underwent ^68^Ga-FAPI-PET/CT at the University Hospital of Heidelberg between July 2017 and July 2020. All patients were referred by their treating oncologists for suspected malignancies, which was confirmed in the majority of cases. Thus, the cohort consists of oncological patients (*n* = 188) with a small number of non-oncological patients (*n* = 11) (Table 1***)***. Written informed consent was obtained from all patients on an individual basis. The retrospective data analysis was approved by the local ethics committee (approval S358/2022).

**Table 1:** Patient characteristics

Patient characteristics	Number of patients
Total number of patients	199
Sex	
Male	114
Female	85
Disease entitiy	
PDAC	43
Head and neck cancer	29
Colorectal cancer	24
Gynecological cancer	19
Lung cancer	12
Prostate cancer	9
Esophageal cancer	8
CUP	7
Sarcoma	6
Thyroid cancer	6
Cholangiocarcinoma	6
Gastric cancer	4
Urothelcarcinoma	2
Liver cancer	1
Melanoma	1
Other malignancies	11
Benign	11

*PDAC* Pancreatic ductal adenocarcinoma, *CUP* Carcinoma of unknown primary

### Image acquisition

All PET scans were performed 1 h after intravenous tracer administration using a Biograph mCT Flow scanner (Siemens, Erlangen, Germany). Imaging data were acquired in 3-dimensional mode (matrix, 220 × 220) with an acquisition time of 3 min per bed position. Attenuation correction was performed using CT data (170 mAs, 100 kV, 2 mm slice thickness). The following FAP ligands were used for FAP imaging: ^68^ Ga-FAPI-02, *n* = 16; ^68^ Ga-FAPI-04, *n* = 138; ^68^ Ga-FAPI-46, *n* = 45. The median injected activity was 192 MBq (range 121–325 MBq). Radiosynthesis and labeling of the FAP tracer was performed at the University of Heidelberg as described previously (Lindner, Giesel, Meyer).

### Image evaluation

Tracer uptake in the liver was quantified using the mean and maximum standardized uptake value (SUVmean and SUVmax). For SUV calculation, circular regions of interest (ROI) of 2 cm diameter were drawn in the liver parenchyma on transaxial slices and automatically fitted to a 3-dimensional volume of interest using Syngovia (Siemens) with a 60% isocontour. The ROI was defined in the region of the liver parenchyma with the most homogeneous appearance. Tracer uptake in the blood pool was measured by placing the ROI of 1 cm diameter in the descending aorta.

### Calculation of fibrosis index

The aspartate aminotransferase (AST) to platelet ratio index (APRI) and the fibrosis index based on 4 factors (FIB-4) were calculated based on the laboratory test results. The maximum time interval of laboratory data and FAP scan was 6 weeks. These values were calculated using the following formulae: APRI = AST level (/upper limit of normal)*100/ platelet count (10^9^/L). FIB-4 = (age*AST)/(platelet count*√ALT) (ALT: alanine transaminase).

### Statistical analysis

Statistical analysis was performed using SigmaPlot version 11.0 (Systat Software, Inc., San Jose, CA, USA). Comparison of tracer uptake between groups was determined using a two-sided t-test. A *P*-value of less than 0.05 was considered as statistically significant. The correlation between tracer uptake and clinical parameters was determined using Pearson correlation analysis. Regression analysis was performed for parameters that correlated significantly with each other. Analysis of variance (ANOVA) using Kruskal–Wallis Test was performed to evaluate the differences in FAPI uptake in the liver between the groups of patients with low (< 0.5), middle (0.5–1.0) and higher (> 1.0) levels of APRI.

## Results

### Baseline characteristics

The cohort included 114 male and 85 female patients with a median age of 63 years (range 16–92 years). 94% of the cohort were oncological patients with different cancer entities (Table 1).

### Correlation between hepatic FAPI uptake liver CT density

**Fig. 1:**
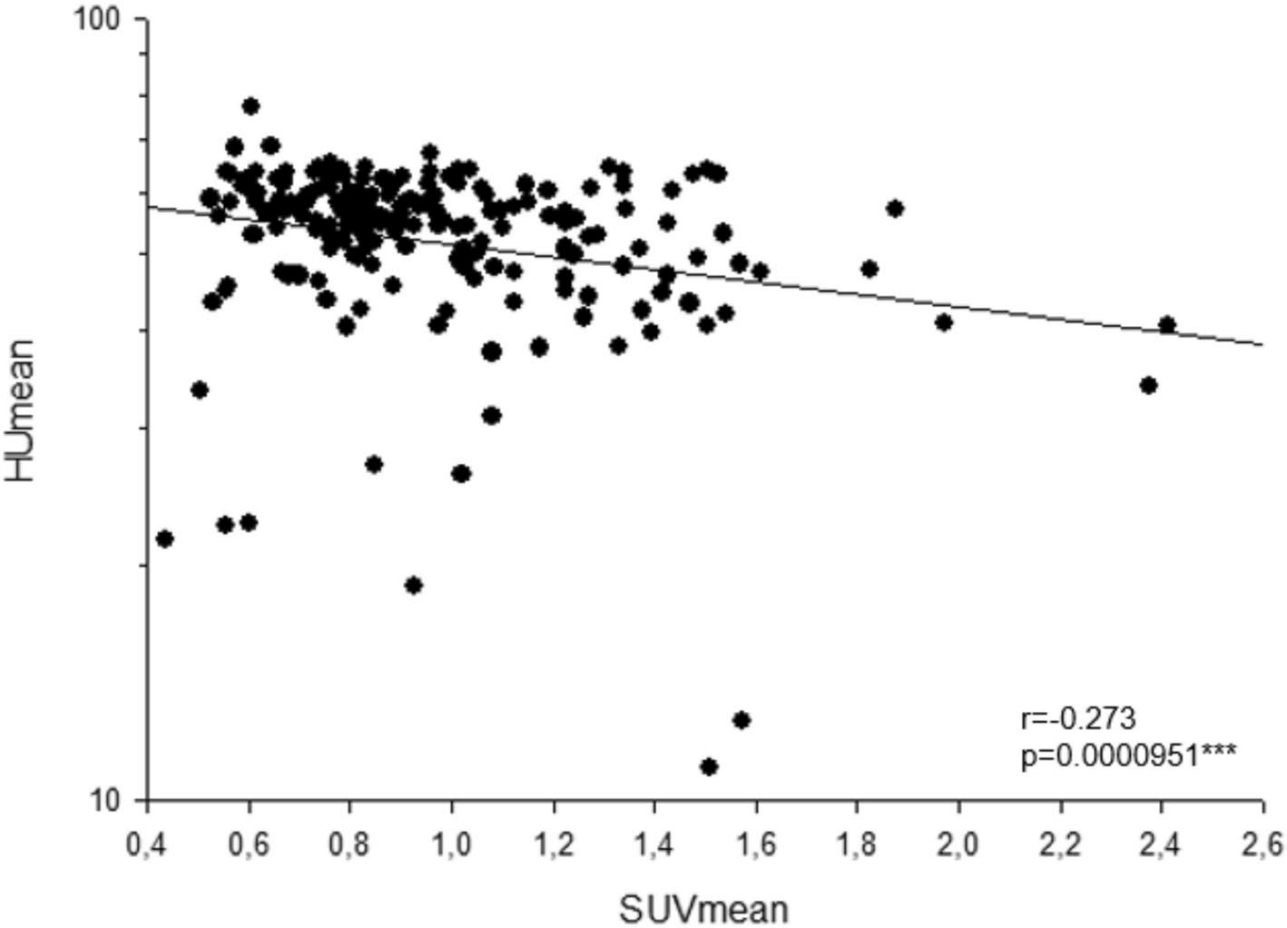
Correlation between liver FAPI uptake and CT density (Hounsfield scale)

FAP ligand uptake in the liver was assessed as SUVmean and SUVmax (*n* = 199). The SUVmean was 0.968 ± 0.331 and the SUVmax 1.592 ± 0.575, respectively. The mean CT density (Hounsfield scale, HUmean) was 52.84 ± 10.92HU. We observed a significant correlation between the SUVmean and the HUmean (*r* = − 0.273, *P* < 0.001***, Fig. 1).

### Correlation between hepatic FAPI uptake and markers of liver fibrosis

**Fig. 2:**
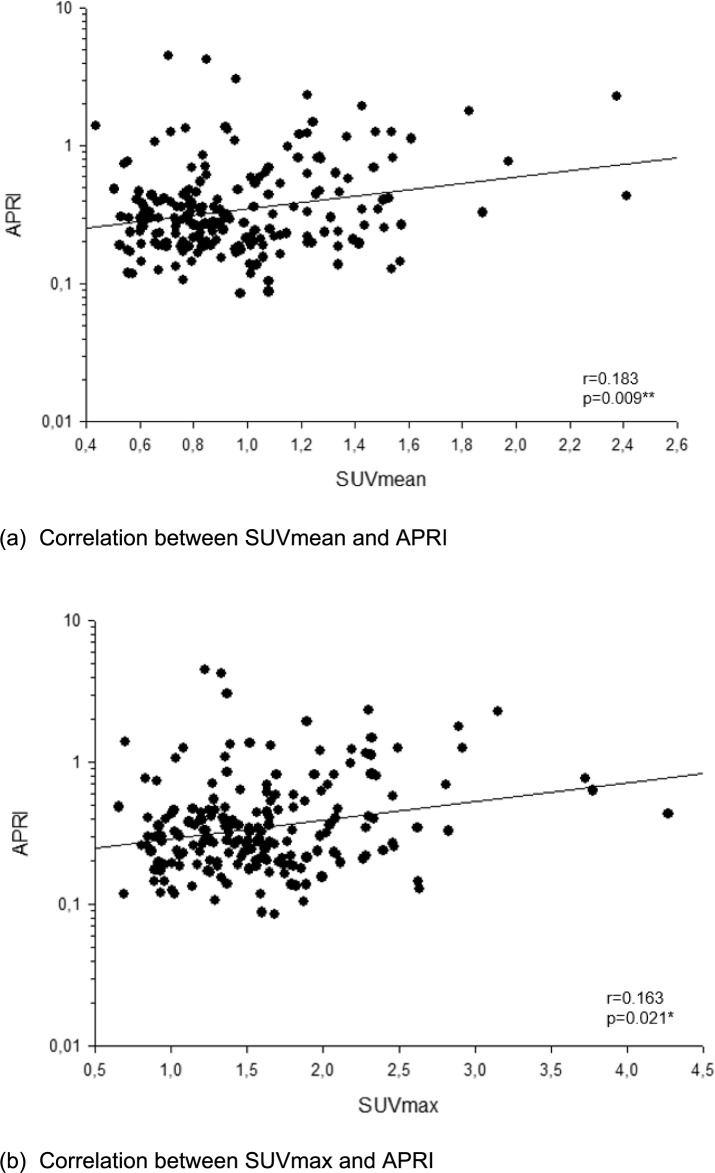
Correlation between liver FAPI uptake and APRI score

**Fig. 3:**
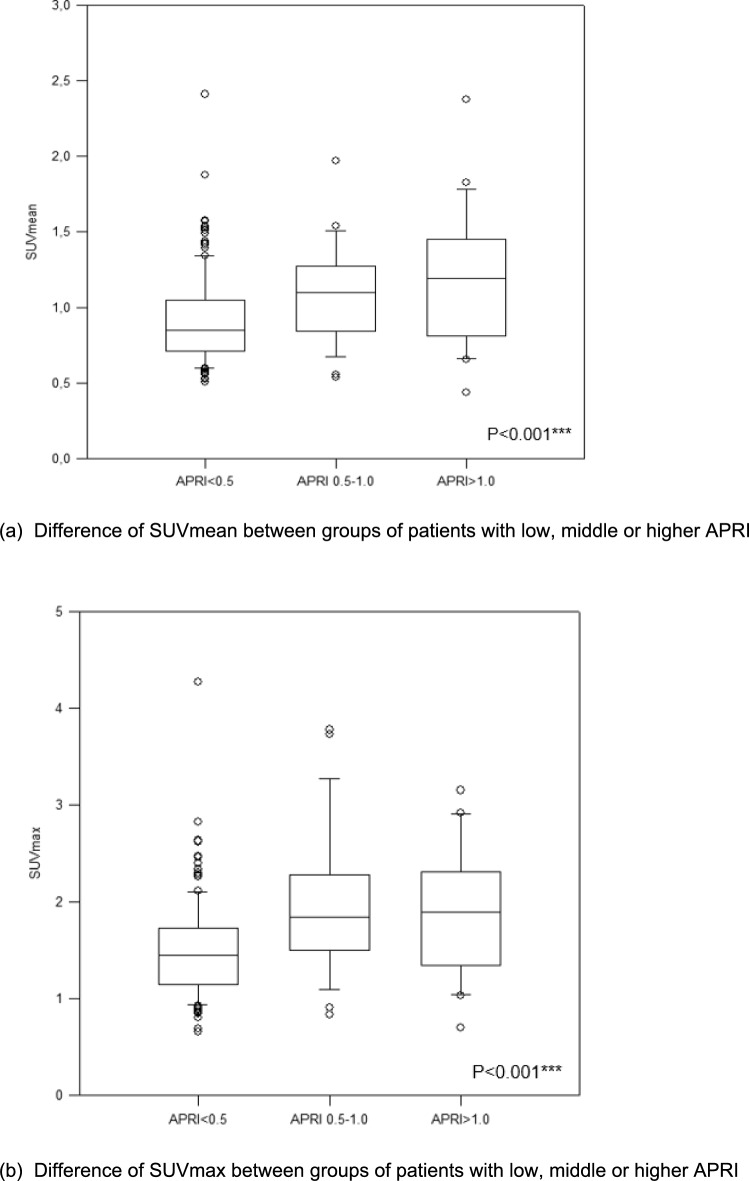
FAP ligand uptake in liver

**Fig. 4:**
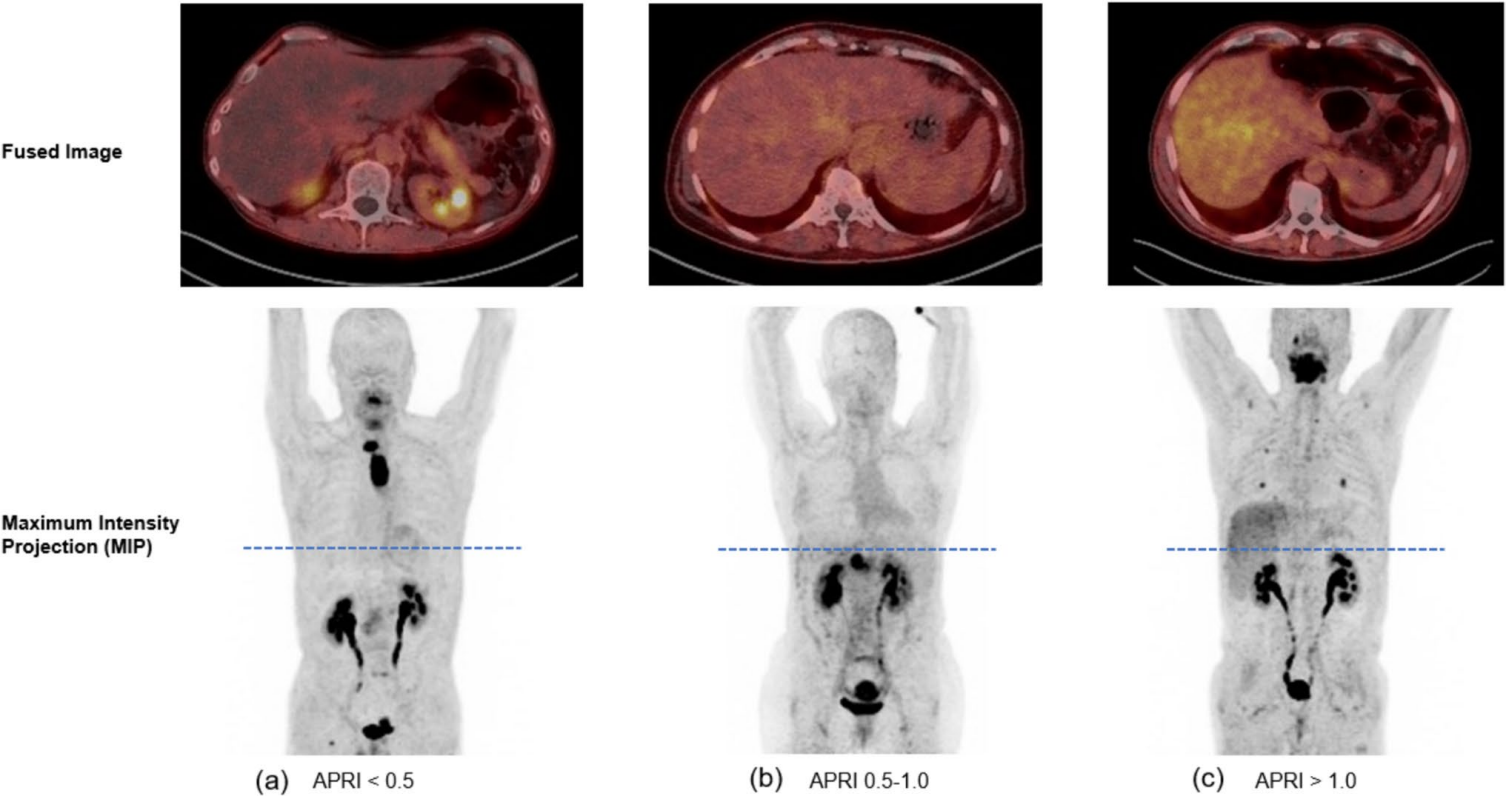
FAP ligand uptake in liver. Representative images of FAPI-PET/CT scan for elevated APRI (**a**), moderately elevated APRI (**b**) and low APRI (**c**)

We next analyzed a potential correlation between the hepatic SUVmax, SUVmean and the two liver fibrosis indices APRI and FIB-4. The median values of APRI and FIB-4 were 0.296 (range 0.080–4.481) and 1.507 (range 0.117–20.101), respectively. There was a significant correlation between both, the SUVmean as well as the SUVmax and the APRI score (*r* = 0.183, *P* < 0.009** and *r* = 0.163, *P* = 0.021*, Fig. 2a–b). Linear regression analysis revealed a regression coefficient of *R* = 0.183 (*P* = 0.010*) and *R* = 0.163 (*P* = 0.02*). There was no significant correlation between hepatic FAPI uptake and the FIB-4 index. We then compared SUVmax and SUV mean values between three subgroups of patients based on their respective APRI score (< 0.5, 0.5–1.0 and > 1.0). Here, we observed a significant and stepwise increase of SUVmean (*P* < 0.001***) as well as the SUVmax (*P* < 0.001***), respectively (Figs. 3–4).

## Discussion

Recent reports on fibrosis imaging using FAP ligands suggest a great potential for imaging and monitoring fibrotic changes in various organs with a simple, repeatable whole-body scan [14–16]. As biopsies are associated with a risk of morbidity, high patient burden and a lack of cost-effectiveness, fibrosis imaging with FAP holds great promise in this regard. There are still insufficient data to assess liver fibrosis, but in a preclinical porcine model, Pirasteh et al. have previously shown that hepatic ^68^ Ga-FAPI-46 uptake strongly correlates with the degree of fibrosis, as indicated by collagen proportionate area (CPA) (*r* = 0.89, *P* < 0.001) [17]. ^68^ Ga-FAPI-46 uptake in this study was significantly and progressively higher with increasing stage of liver fibrosis (*P* < 0.001) [17], which is corroborated in another study using a preclinical mouse model and subsequent human translation, evaluating 26 patients with confirmed liver fibrosis [18]. This translational study showed a correlation between ^68^ Ga-DOTA-FAPI-04 uptake and fibrosis stage (*r* = 0.653 to 0.698, all *P* < 0.01) [18]. The strong correlation between liver ^68^ Ga-FAPI-46 uptake and the histological stage of liver fibrosis suggests that FAPI-PET may play an important role in the non-invasive staging of liver fibrosis, which may be pathophysiologically explained by the fact that activated hepatic stellate cells (HSCs) express FAP and are thought to play an essential role in promoting fibrosis in the liver. HSCs, which in the quiescent state represent 5–10% of the total number of liver cells, begin to proliferate and differentiate into myofibroblasts upon paracrine stimulation by neighbouring cells, including Kupffer cells, hepatocytes or sinusoidal endothelial cells [19].

To date, several methods have been proposed to non-invasively assess the severity of liver fibrosis [20]. These include radiographic assessment, stiffness measurement (liver elastography), and several scoring systems based on laboratory tests, although all these mentioned methods remain still controversial. In radiology, the iodine density of the liver parenchyma in relation to that of the aorta, obtained from the equilibrium phase on dynamic contrast-enhanced CT, is reported to be useful for the staging of liver fibrosis [21], while other authors demonstrated the significant predictive value of the iodine washout rate (IWR), calculated from the hepatic iodine uptake during the portal venous phase (PVP) and the 3 min delayed phase (DP) using multiphasic dual-energy CT [22]. Liver elastography offers the possibility of a rapid, non-invasive, and painless assessment of the liver with several options available, e.g. transient elastography, point shear wave elastography, 2D shear wave elastography, or magnetic resonance elastography [23, 24]. However, patient, operator and examination characteristics have all been shown to influence the result of liver stiffness measurements, e.g. food intake increases liver stiffness, whereas alcohol withdrawal is associated with a decrease in elastography results. The inter-observer reproducibility of the measurement seems suboptimal, and the influence of the operator experience is still being debated [23]. Regarding scoring systems, several scores have been proposed and are widely used in clinical routine for the non-invasive assessment of fibrosis due to their easy availability [25, 26]. Aspartate aminotransferase (AST)-to-platelet ratio index (APRI) and the fibrosis index based on 4 factors (FIB-4) are the two commonly used indices in chronic liver disease [27], but the performance of these indices remain controversial [28–31]. It has been suggested that they may overestimate the fibrosis stage due to the effect of necroinflammatory activity on transaminases in chronic hepatitis [27, 32]. Another limitation appears to be the limited sensitivity especially for fibrosis in advanced stage [33, 34]. In a meta-analysis comparing the performance of APRI and FIB-4 in patients with hepatitis B, revealed for APRI the sensitivity and specificity of 70% and 60%, 50% and 83%, and 36.9% and 92.5% for mild fibrosis, advanced fibrosis, and cirrhosis, respectively (APRI thresholds: 0.5, 1, and 1.5) and for FIB-4 the sensitivity and specificity of 65.4% and 73.6%, 16.2% and 95.2% for mild and advanced fibrosis (FIB-4 thresholds 1.45 and 3.25, respectively) [33]. In another study evaluating patients with hepatitis C, APRI showed similar performance to FIB-4 with a positive predictive value (PPV) of 77% (for APRI > 1.5) and a negative predictive value (NPV) of 83% (for APRI < 0.5) [34]. A cutoff of 0.5 (APRI) showed 81% of sensitivity and 50% of specificity, while a cutoff of 1.5 was more specific (94%) and less sensitive (42%) in this study [34]. This suggests that at least APRI is not sensitive enough to detect advanced fibrosis, but probably suitable to exclude healthy patients in the early stage. For FIB-4, a large cross-sectional study enhancing 5129 patients revealed that almost one-third (28%) of elevated FIB-4 was false-positive [35].

In view of this insufficient situation, we hypothesized that FAP imaging might be useful as a non-invasive imaging method for the assessment of liver fibrosis. The basic characteristic of our present study to be considered in the interpretation of our results is that our cohort consists of patients who were originally referred for FAPI-PET/CT due to suspected malignancy of any etiologies. Thus, the basic character of the cohort is somewhat similar to that of a general population in the respect that no previous selection of patients was performed due to the known liver pathologies. This matches the resulting overall low to moderate hepatic FAPI uptake and majorly normal liver enzymes levels in our results.

In the current study, we found a strong negative correlation between hepatic FAPI uptake and CT density (Hounsfield scale). This may be possibly due to the fact that lipogenic alteration of liver parenchyma is one of the most frequent phenomenon in the initial phase of fibrotic liver processes, the most common causes being alcoholic and non-alcoholic fatty liver diseases. Further, we found that hepatic FAPI uptake correlates weak but significantly with APRI. Based on this result, we split in the next step the patients into three groups according to the level of APRI. This resulted in a significant difference in SUV value between the groups with a weak positive correlation. Interestingly, FIB-4 showed no correlation with the uptake value of FAP ligand in the liver. The possible interpretation of these results is that APRI may possibly show better performance in detecting early fibrotic changes compared to FIB-4, although both scoring systems do not seem to be sensitive enough to detect advanced fibrosis, as mentioned above. For the conclusive analysis of the performance of FAPI-PET though, a histological validation is essential, which is not available in this retrospective study.

There are several essential limitations in the present study. The most significant limitation is the lack of histology as already mentioned, for the ultimate validation of the accuracy of each method. Another main limitation is the character of the cohort with non-selective benign and malignant diseases. Although this might partly provide an advantage to mimic a general population cohort for screening, it seems yet to limit the validity of our results essentially, because the majority of patients have no pathologic elevation of liver enzymes or platelet counts. Other limitations include varying ^68^ Ga-FAP tracers and the time interval between FAP imaging and laboratory tests.

## Conclusion

FAP imaging is possibly an effective method for the non-invasive detection of liver fibrosis especially in the early phase, which is frequently accompanied by lipogenic changes and slightly altered serum parameters. Although the currently presented data are promising, further evaluation in a selected patient cohort with histological validation and a well-designed preclinical study with various liver pathologies are necessary to determine the accuracy of the best surrogate marker for liver fibrosis.

## Supplementary Information

Below is the link to the electronic supplementary material.

Supplementary file1 (DOCX 440 KB)

## Data Availability

The data used and/or analyzed during the current study are available from the corresponding author on reasonable request.
