# Supplementary material for: Efficacy of [68Ga]Ga-FAPI-PET as a non-invasive evaluation method of liver fibrosis
Source: Ann Nucl Med. 2025 Mar 6;39(6):631–9. doi: 10.1007/s12149-025-02027-6 (PMC12095406; doi:10.1007/s12149-025-02027-6)
Supplement: Supplementary file 1 — Supplementary file1 (DOCX 440 KB) [file 12149_2025_2027_MOESM1_ESM.docx]

**Supplement:**


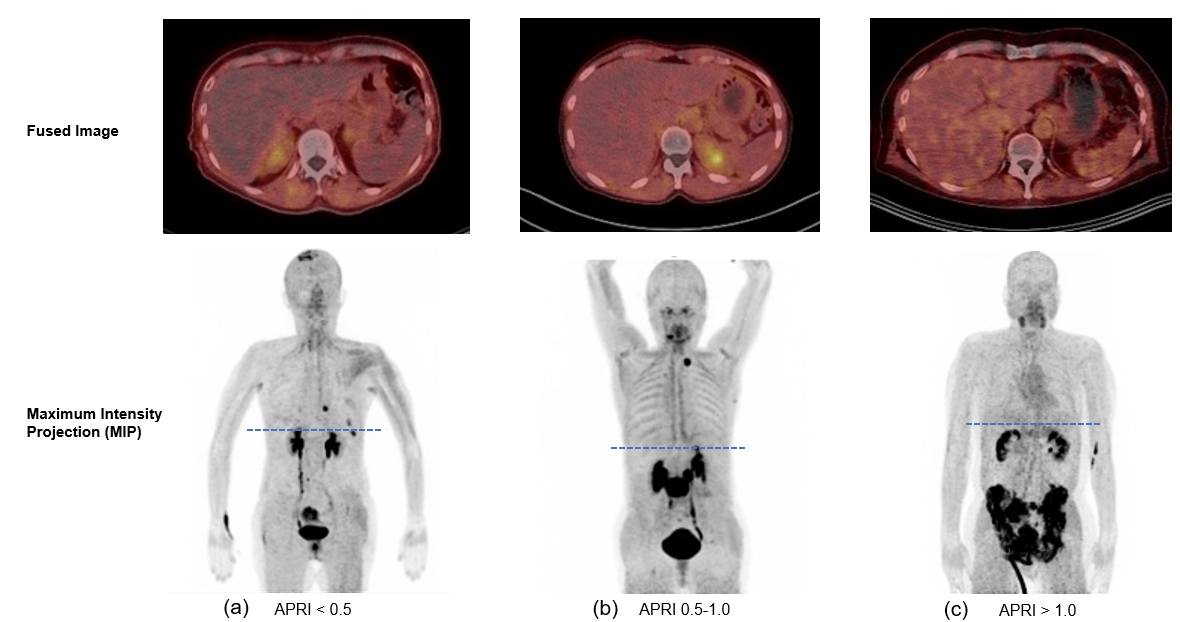


Supplement Figure 1: Additional images of FAPI-PET/CT scan for lower APRI (a: 59-year-old female patient with glioblastoma; SUVmean 0.590, APRI 0.407), moderate APRI (b: 29-year-old female patient with uterus cancer; SUVmean 0.845, APRI 0.707) and elevated APRI (c: 54-year-old male patient with prostate cancer; SUVmean 1.225, APRI 2.330).
